# The predictive value of peripheral blood CD4 cells ATP concentration for immune-related adverse events in advanced non-small cell lung cancer patients

**DOI:** 10.1186/s12865-023-00592-x

**Published:** 2024-01-06

**Authors:** Kailian Hou, Weipeng Ye, Qunfeng Huang, Weiyi Li, Zhiqiong Tan, Na tao, Dongheng yang, Haoxin Lin, Zihao Deng, Yuanyuan Xia, Guifang Yu

**Affiliations:** Department of Oncology, The Fifth Affiliated Hospital of Guangzhou Medical University, No. 621, Gangwan Road, Huangpu District, Guangzhou, 510700 China

**Keywords:** Non-small cell lung cancer, CD4 cells, Adenosine triphosphate, Immune-related adverse events

## Abstract

**Objective:**

Lung cancer with the highest incidence and mortality in the world. Immune checkpoint inhibitors (ICIs), can bring long-term survival benefits to patients, but also can bring immune-related adverse events (irAEs) in some patients during therapy. Therefore, the aim of this study was to investigate the predictive effect of peripheral blood WBC, NLR, sATP^CD4^ and nATP^CD4^ on irAEs in advanced non-small cell lung cancer (NSCLC).

**Methods:**

Clinical data of 112 patients with advanced NSCLC who were treated with PD -1/PD -L1 inhibitor in the Fifth Affiliated Hospital of Guangzhou Medical University from December 15, 2019 to April 30, 2023 were retrospectively analyzed. These patients were divided into the irAEs group (n = 27) and non-irAEs group (n = 85). The clinical data of the two groups were compared. Receiver operating characteristic (ROC) curves were drawn to determine the threshold value of baseline peripheral blood parameters to predict the occurrence of irAEs. Multivariate logistic regression analysis was used to explore the relationship between peripheral blood markers and the incidence of irAEs.

**Results:**

The patient characteristics have no significant difference between irAEs and non-irAEs group. But the baseline peripheral blood WBC, sATP^CD4^ and nATP^CD4^ of patients in the irAEs group were higher than those in the non-irAEs group (*p* < 0.05), and the NLR in irAEs group was similar to in the non-irAEs group (*p* = 0.639).Univariate analysis showed that high WBC, sATP^CD4^ and nATP^CD4^ may the risk factors for the occurrence of irAEs (*p* < 0.05). Multivariate logistic regression analysis showed that high sATP^CD4^ and nATP^CD4^ were independent risk factors for the occurrence of irAEs (*p* < 0.05). The best critical values of WBC, sATP^CD4^ and nATP^CD4^ before treatment for predicting the occurrence of irAEs were 8.165 × 10^9^cells/L (AUC = 0.705) ,484.5 ng/mL (AUC = 0.777), and 156 ng/mL (AUC = 0.840), respectively.

**Conclusions:**

sATP^CD4^ and nATP^CD4^ were independent risk factors for the occurrence of irAEs in advanced NSCLC patients. This discovery provides a new method to predict the occurrence of irAEs in patients. Based on the prediction results, corresponding treatment measures can be taken to reduce the incidence of adverse events.

## Introduction

Lung cancer is the most prevalent and lethal tumor globally, with non-small cell lung cancer (NSCLC) accounting for approximately 80% of cases [1]. In recent years, immune checkpoint inhibitors (ICIs), such as antibodies against programmed cell death-1 (PD-1), programmed cell death-ligand 1 (PD-L1), and cytotoxic T-lymphocyte-associated protein-4 (CTLA-4), have demonstrated remarkable clinical efficacy in lung cancer treatment by enhancing the immune system’s ability to combat tumor cells [2]。While ICIs provide patients with long-term survival benefits, the associated immune-related adverse events (irAEs) cannot be ignored [3, 4].Studies have shown that the incidence of irAEs ranges from 15 to 90%, with the proportion of severe irAEs requiring drug intervention ranging from 0.5 to 13%. Additionally, 43% of patients receiving combination therapy require treatment termination due to adverse events. Therefore, early recognition, management, and care of irAEs are crucial for the safe use of ICIs [3, 4].

Unlike traditional radiation therapy, chemotherapy, or targeted therapy-related toxicity, irAEs are not directly caused by drug action. Instead, they result from drug-induced autoimmune abnormalities or inflammatory reactions [5]. Infammation is closely linked to cancer, as it promotes a favorable microenvironment for cancer cell growth and spread, and activation of carcinogenic signaling pathways [6, 7]. The prognostic value of some infammation-related peripheral blood parameters has been investigated, including the neutrophil-to-lymphocyte ratio (NLR), lactate dehydrogenase (LDH), and prognostic nutritional index (PNI). Calculation of the NLR depends on the absolute neutrophil count and the absolute lymphocyte count within the peripheral blood; some studies have shown that NLR is associated with worsened prognosis in patients with melanoma [8–10] and NSCLC [11] receiving immunotherapy.

Alexander X Lozano found that the distribution pattern and function of CD4^+^ cells are closely related to the occurrence of irAEs [12, 13]. Akiko Arakawa et al. found that diverse T-cell clones in the blood of melanoma patients prior to immunotherapy, which may reflect the extent to which T cells are able to react against melanoma and potentially control melanoma progression [14]. Therefore, the T-cell function may have predictive value for antitumor responses and irAEs from checkpoint inhibition.

The CD4^+^ cell Adenosine triphosphate (ATP) release assay measures the concentration of ATP within purified CD4^+^ T lymphocytes in vitro, either stimulated by phytohemagglutinin (PHA) to obtain stimulated ATP release (sATP^CD4^) or without stimulation to measure non-stimulated ATP release (nATP^CD4^). This assay has been developed to evaluate cell-mediated immune function, predict infection risk, and assess organ transplant rejection after solid organ transplantation [13, 14].

The aim of this article is to explore the predictive value of peripheral blood parameters, including WBC, NLR, sATP^CD4^, and nATP^CD4^, for irAEs in advanced NSCLC.

## Materials and methods

### Patients

This retrospective study collected data from December 15, 2019, to April 30, 2023, involving 112 patients with advanced non-small cell lung cancer (stage IIIB/IV) who underwent PD-1/PD-L1 inhibitor treatment at the Fifth Affiliated Hospital of Guangzhou Medical University. The patients were staged according to the 8th edition TNM staging system. Inclusion criteria encompassed patients who received PD-1/PD-L1 inhibitor treatment for the first time, had complete follow-up information, and baseline peripheral blood sample test data. To capture potential irAEs occurring within the initial 12 weeks, a minimum follow-up period of 3 months was required for study inclusion. Exclusion criteria consisted of patients with autoimmune disease, pulmonary interstitial disease, adrenal insufficiency, or systemic immunosuppression. This study was conducted with approval from the Fifth Affiliated Hospital of Guangzhou Medical University Ethics Committee (Approval No. KY01-2022-07-08).

### Treatment and data collection

Clinical characteristics of the patients, such as age, gender, histology, and sensitive gene mutation status, were recorded. Baseline measurements were defined as those taken within 1 week prior to the administration of PD-1/PD-L1 inhibitors. The baseline peripheral blood data including WBC (white blood cell count), NLR value (absolute neutrophil count divided by absolute lymphocyte count) and a CD4 cells ATP release assay (includes nATP^CD4^ and sATP^CD4^).

### Study assessments

The assessment of irAEs will span a 12-week period, as previous research has shown that the highest incidence of irAEs typically transpires within this timeframe. IrAEs encompass adverse events that indicate an immune system dysfunction, including but not limited to rash, colitis, liver dysfunction, thyroid disorder, and other related conditions.

### CD4^+^ cells ATP release assay

The intracellular ATP concentration produced by CD4^+^ cells were measured by luciferin-luciferase reaction both after stimulation with or without mitogen PHA, as described in the manufacturer’s instructions (AIMdex, Leide Biosciences Co., Ltd, China, Guangzhou). Briefly, 100µL of whole blood was diluted 4-fold and incubated with or without 25µL PHA(8.75ng/ml) for 15 to 18 h in a 5% carbon dioxide incubator at 37 ± 0.5℃. CD4^+^ cells were subsequently separated and purified using magnetic beads conjugated with monoclonal antibodies specific for CD4^+^ cells. To release ATP, a lysis buffer was added to the cells. Following that, a luciferin/luciferase mixture was introduced to the cell lysate and thoroughly mixed. The bioluminescent product was then measured within 30 min using a luminometer (JR-I, Weihai Weigao Biotechnology Co., LTD.). For the development of an ATP standard graph, various aliquots (0, 50, 100, 200, 400, and 800 ng/mL) of ATP calibrators were employed. The ATP levels were compared between CD4^+^ cell-depleted and non-depleted samples to ensure that the readings were specific to ATP produced by the isolated cells.

### Statistical analysis

Statistical analysis was conducted using SPSS 20.0. For categorical variables in the clinical and demographic data, the chi-squared test, Student’s t-test, and Mann-Whitney U test were employed to compare differences between categorical variables, normally distributed continuous variables, and non-normally distributed continuous variables, respectively. Logistic regression analysis was utilized to ascertain the correlation between peripheral blood biomarkers and the occurrence of irAEs. ROC curves were employed to determine the critical values of baseline peripheral blood parameters.

## Results

### Patient characteristics and treatment

In our study, a total of 112 patients were included in the final analysis (Table 1). All patients were divided into irAE (n = 27) and non-irAE groups (n = 85). There were no significant differences in gender (*p* = 0.455, χ^2^ = 0.559), age (*p* = 0.483, χ^2^ = 0.492), BMI (*p* = 0.338, χ^2^=-0.862), smoking history (*p* = 0.313, χ^2^ = 1.016), ECOG-PS (*p* = 0.426, χ^2^ = 0.634), Histology (*p* = 0.420, χ^2^ = 0.649), Line of treatment with ICIs (*p* = 0.994, χ^2^ = 0.000), Gene mutation (*p* = 0.365, χ^2^ = 0.821), Treatment data (*p* = 0.550, χ^2^ = 1.195) between irAEs and non-irAEs groups.

**Table 1:** Comparison of baseline characteristics between irAEs and non - irAEs groups

variables	irAEs (n = 27)	non-irAEs (n = 85)	χ^2^/Z	*p* value
gender			0.559	0.455
male	14(51.9%)	51(60%)		
female	13(48.1%)	34(40%)		
age (years)			0.492	0.483
< 60	10(37.0%)	38(44.7%)		
≥ 60	17(63.0%)	47(55.3%)		
BMI	20.9122 ± 1.91697	21.6695 ± 3.10683	-0.862	0.388
smoking history			1.016	0.313
yes	7(25.8%)	31(36.5%)		
no	20(74.1%)	54(63.6%)		
ECOG-PS			0.634	0.426
0–1	26(96.3%)	77(91.8%)		
≥ 2	1(3.7%)	7(8.2%)		
Histology			0.649	0.420
Adenocarcinoma	24(88.9%)	70(82.4%)		
Squamous carcinoma	3(11.1%)	15(17.3%)		
Line of treatment with ICIs			0.000	0.994
1	14(51.9%)	44(51.8%)		
≥ 2	13(48.1%)	41(48.2%)		
Gene mutation			0.821	0.365
Yes	8(29.6%)	18(21.2%)		
No	19(70.4%)	67(78.8%)		
Treatment data			1.195	0.550
Monotherapy	3(11.1%)	12(14.1%)		
Combination with chemotherapy	21(77.8%)	57(67.1%)		
Combination with other treatments	3(11.1%)	16(18.8%)		

irAEs, immune-related adverse events; BMI, Body Mass Index; ECOG PS, Eastern Cooperative Oncology Group performance status; ICIs, immune checkpoint inhibitors

### Comparison of peripheral blood indicators between irAEs and non-irAEs

**Fig. 1:**
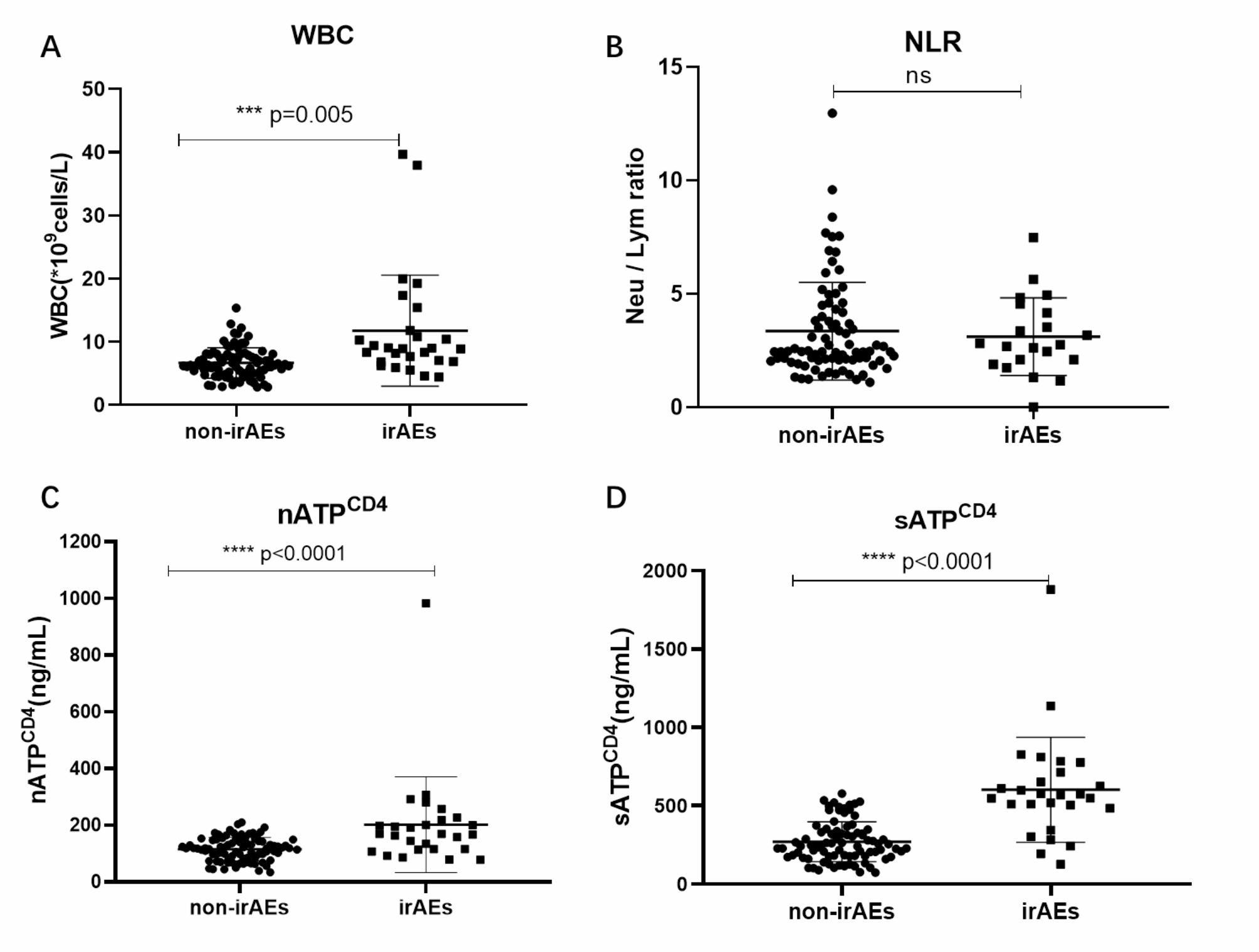
Comparison of peripheral blood indicators between irAEs and non - irAEs groups. **(A)** WBC between irAEs (n = 27) and non - irAEs groups (n = 85) ; **(B)** NLR between irAEs and non - irAEs groups; **(C)** nATP^CD4^ concentration between irAEs and non - irAEs groups; **(D)** sATP^CD4^ concentration between irAEs and non - irAEs groups; irAEs,immune-related adverse events; WBC, white blood cell; NLR, Neutrophil-to-lymphocyte ratio; nATP^CD4^, Non-stimulated CD4^+^ cells ATP concentration; sATP^CD4^, Stimulated CD4^+^ cells ATP concentration; ***, *p* < 0.005;****, *p* < 0.0001; ns, no significant

The study analyzed immune cell in peripheral blood from 112 patients (Fig. 1). The results showed that WBC was significantly higher in irAEs compared to non-irAEs (*P* = 0.005) (Fig. 1A). However, no significant differences were observed in the NLR between the irAEs and non-irAEs groups (*P* = 0.639) (Fig. 1B). Additionally, in line with the WBC trend, the analysis of nATP^CD4^ and sATP^CD4^ concentrations demonstrated significantly higher levels in patients who developed irAEs compared to those without irAEs (*P* < 0.0001) (Fig. 1C, D).

### Subtypes of irAEs in the study population

The incident rate of irAEs was 24.11% (27/112). Of the 27 patients with irAEs, 44.44%(12/27) had grade 1 irAEs, 37.04% (10/27) had grade 2 irAEs, and 22.22% (6/27) had grade 3 irAEs. The specific details of irAEs were as follows: 8 patients (29.6%) had skin responses, most of which were of grade 1–2(75%) with rash being the most common irAEs; 7 patients (25.9%) had endocrine irAEs, of whom 5 were grade 1–2 AEs, and 2 patients had grade 3 irAEs. The incidence rates of gastrointestinal adverse reactions, hematological adverse reactions, immune-associated pneumonia, immune-related liver injury, and other irAEs were 11.1%(3/27), 3.7%(1/27),18.5%(5/27), 7.5%(2/27), and 3.7%(1/27), respectively (Table 2).

### Analysis of predictors of irAEs

**Fig. 2:**
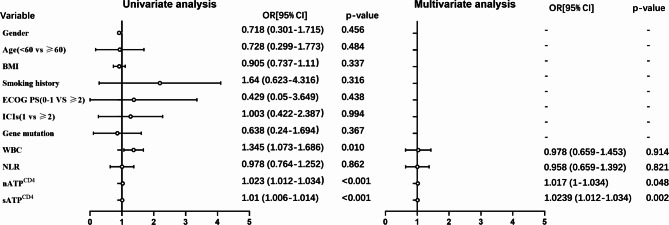
Univariate and multivariable analysis of irAEs. Variables with *p* ≤ 0.05 in univariate models were analyzed in the multivariate analysis model (Many studies have shown a close association between NLR and irAEs. Therefore, this study included NLR in the multivariate analysis to further investigate its relationship). BMI, Body Mass Index; ECOG PS, Eastern Cooperative Oncology Group performance status; ICIs, immune checkpoint inhibitors; WBC, white blood cell; NLR, Neutrophil-to-lymphocyte ratio; nATP^CD4^, Non-stimulated CD4^+^ cells ATP concentration; sATP^CD4^, Stimulated CD4^+^ cells ATP concentration; OR, Odds ratio; CI, Confidence interval

The relationship between the tested variables and irAEs was examined using both univariate and multivariate logistic regression analyses. The univariate analysis revealed that WBC count (*p* = 0.01), sATP^CD4^ (*p* < 0.001), and nATP^CD4^ (*p* < 0.001) were significantly correlated with the occurrence of irAEs. However, factors such as gender, age, smoking status, ECOG PS score, the specific line of treatment with immune checkpoint inhibitors (ICIs), gene mutation, treatment data, and NLR did not show a significant correlation with irAEs. The results of the multivariate analysis in the WBC, nATP^CD4^, sATP^CD4^and NLR indicators were consistent with the findings observed in the overall study population. Furthermore, high nATP^CD4^ (*p* = 0.048) and sATP^CD4^ (*p* = 0.002) concentration were identified as independent risk factors associated with irAEs (Fig. 2).

**Table 2:** Subtypes of irAEs in the study population n (%)

Subtypes of irAEs	Total	Grade		
		1	2	3
irAEs	27(24.11%)	12(44.40%)	10(37.04%)	6(22.22%)
Adverse skin reaction	8(29.6%)	3(37.50%)	3(37.50%)	2(25.00%)
Adverse endocrine reaction	7(25.9%)	3(42.90%)	2(28.60%)	2(28.60%)
Gastrointestinal adverse reactions	3(11.1%)	2(66.70%)	1(33.30%)	0(0.00%)
Hematology adverse reaction	1(3.7%)	0(0.00%)	1(100.00%)	0(0.00%)
Immune associated pneumonia	5(18.5%)	1(20.00%)	2(40.00%)	2(40.00%)
Immune - related liver injury	2(7.5%)	1(50.00%)	1(50.00%)	0(0.00%)
Others	1(3.7%)	1(100%)	0(0.00%)	0(0.00%)

irAEs, immune-related adverse events

### Cut-off points and predictive values

**Fig. 3:**
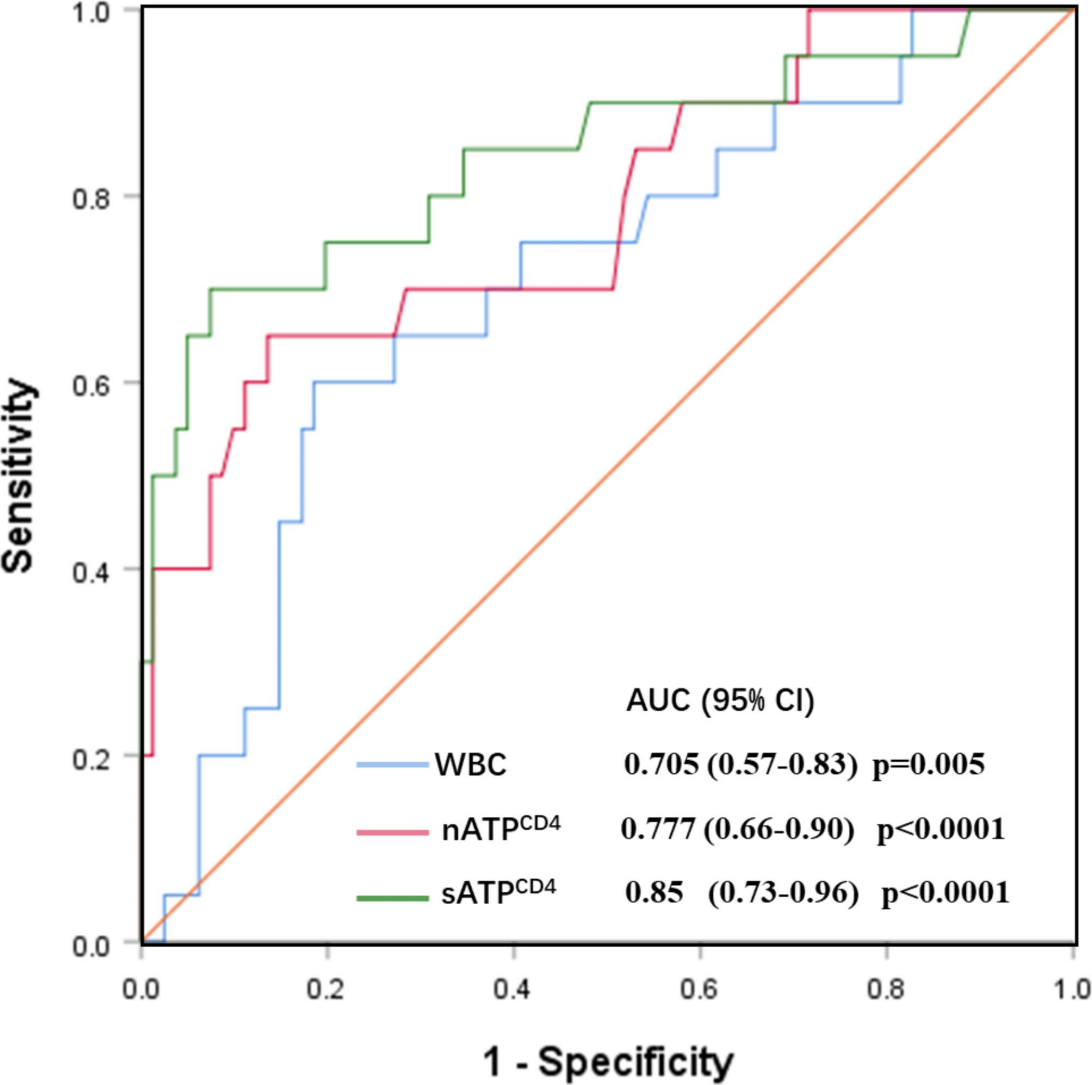
ROC curves of irAEs predicted by WBC, nATP^CD4^ and sATP^CD4^. AUC, area under curve; WBC, white blood cell; nATP^CD4^, Non-stimulated CD4^+^ T lymphocytes ATP concentration; nATP^CD4^, Non-stimulated CD4^+^ cells ATP concentration; sATP^CD4^, Stimulated CD4^+^ cells ATP concentration; CI, Confidence interval

To determine the cut-off points for the occurrence of irAEs, ROC analyses were conducted (Fig. 3). The sATP^CD4^ concentration of 484.5 ng/ml (area under the curve (AUC) = 0.840, 95% CI: 0.726–0.955), specificity 62.7% and sensitivity 70.0%) were identified as best cut-off value for predicting risk of irAEs, Similarly, the nATP CD4 levels of 156 ng/mL (AUC = 0.777, 95% CI: 0.656–0.899, specificity 50.4%, and sensitivity 65.0%) were determined as the best cut-off points for the occurrence of irAEs. The best critical values of WBC before treatment for predicting the occurrence of irAEs were found to be 8.165 × 10^9^cells/L (AUC = 0.705,95% CI: 0.656–0.899, specificity 41.7% and sensitivity 60.0%).

## Discussion

In recent years, ICIs have emerged as a significant advancement in the treatment of advanced NSCLC [15]. However, the use of ICIs in treatment can lead to irAEs. While some irAEs such as rash, thyroid dysfunction, colitis, and diarrhea are typically manageable and treatable, there are certain severe or rare irAEs that can have a significant impact on the effectiveness and progression of immunotherapy in patients [16]. The occurrence of irAEs in clinical practice is personalized, complex, and unpredictable, and in some cases, irAEs can even be life-threatening [17].

Therefore, we conducted a retrospective analysis of patients with advanced NSCLC who received various ICIs to comprehensively evaluate the incidence and toxicity profiles of irAEs in real-world clinical practice. The overall incidence rate of irAEs in our study was found to be 24.1%. Among these cases, grade 1, grade 2, and grade 3 irAEs accounted for 44.4%, 37.4%, and 22.2% respectively. Adverse skin reactions and adverse endocrine reactions were the most commonly observed types of irAEs, with incidence rates of 29.6% and 29.5% respectively. Consistent with previous report [14], the majority of patients showed good recovery through appropriate management and treatment. Notably, two cases of grade 3 immune-related pneumonitis were identified, leading to temporary treatment interruption due to severe symptoms in order to improve the patients’ condition.

ICIs works by reactivating the body’s own immune system to enhance anti-tumor immune responses. However, in doing so, it may inadvertently harm healthy tissues throughout the body, leading to a variety of toxic side effects across different systems. Despite the vigorous research efforts in recent years, the exact pathophysiological mechanisms behind irAEs remain unclear. Current theories suggest that irAEs could be associated with disturbances in peripheral immune tolerance [18], aberrant activation of T cells, increased pro-inflammatory activity of cytokines [19], dysbiosis of gut microbiota [20], recognition of shared antigens by specific autoreactive T cell clones [21] and cross-reactivity between T cells responding to tumor and normal tissue antigens [22]. Although preliminary observations have been made, large-scale clinical data are still needed to further clarify the causes of irAEs.

To identify potential predictive indicators for irAEs, we conducted both univariate and multivariate analyses on multiple factors, including clinical baseline characteristics, WBC, NLR, nATP^CD4^, and sATP^CD4^ obtained one week prior to immunotherapy. Our findings revealed that WBC, nATP^CD4^, and sATP^CD4^ were significantly elevated in the irAEs group compared to the non-irAEs group. Univariate analysis demonstrated a correlation between WBC, nATP^CD4^, sATP^CD4^, and the occurrence of irAEs. Furthermore, multivariate analysis confirmed that high concentrations of nATP^CD4^ and sATP^CD4^ served as independent risk factors for the development of irAEs. These results align with previous research on clinical baseline characteristics, WBC counts, and NLR [23, 24].

The inclusion of nATP^CD4^ and sATP^CD4^ in our study was motivated by existing research indicating that sATP^CD4^ can effectively assess the immune function of organ transplant recipients and predict the occurrence of infections and rejections [25, 26]. Notably, a study on liver transplantation demonstrated a strong correlation between low sATP^CD4^ concentrations and a higher risk of liver cancer recurrence, as well as reduced progression-free survival and overall survival rates [27]. Adjusting clinical protocols based on sATP^CD4^ values has been shown to help decrease infection rates and improve overall survival in liver transplant patients [28, 29], validating ATP^CD4^ as a marker for immune function assessment. Although many studies have indicated a close relationship between the development of immune-related adverse events (irAEs) and the immune function of the body, there has been a lack of research exploring the direct relationship between ATP^CD4^ levels and irAEs. Our study pioneers this area of research, finding for the first time that elevated levels of ATP^CD4^ are independent risk factors for the development of irAEs. This discovery suggests that monitoring nATP^CD4^ and sATP^CD4^ concentrations in peripheral blood prior to treatment could be instrumental in predicting the likelihood of irAEs in advanced NSCLC patients who are undergoing therapy with PD-1/PD-L1 inhibitors.

Furthermore, we observed that high concentrations of nATP^CD4^ were also independent risk factors for the occurrence of irAEs. This suggests that monitoring these peripheral blood markers before treatment could aid in predicting the likelihood of irAEs in advanced NSCLC patients undergoing PD-1/PD-L1 inhibitor therapy.

The results of the ROC curve analysis demonstrated an area AUC of 0.84 for sATP^CD4^ and 0.78 for nATP^CD4^, indicating their potential as predictive indicators for irAEs. Using a cutoff value of 484.5 ng/ml for sATP^CD4^, the sensitivity and specificity were determined to be 60% and 70% respectively. Similarly, with a cutoff value of 156 ng/ml for nATP^CD4^, the sensitivity and specificity were found to be 50% and 60% respectively. The selection of these predictive indicators and the establishment of cutoff values are valuable for understanding the risk factors associated with irAEs. These findings can assist clinicians in optimizing treatment strategies and implementing appropriate monitoring and management protocols for patients undergoing immunotherapy [30, 31]. By adopting a more individualized approach to therapy, balancing the potential benefits of ICIs with the risks of developing irAEs becomes possible. Some studies suggest that a better understanding of immunotherapy at the cellular and molecular levels, coupled with insights into epigenetic modifications, could be utilized to enhance the potential of mitigating irAEs associated with immunotherapy. Epigenomic modifications can alter cellular contextual transcription through ATP-dependent nucleosomal repositioning, thereby influencing cellular immune function [32]. further exploration can be conducted on the correlation between epigenetic modifications and CD4^ATP^, as well as their predictive value for the efficacy of immunotherapy and irAEs. By delving deeper into these associations, we can gain a better understanding of the mechanisms behind immunotherapy and provide more accurate guidance for personalized treatment.

It is important to acknowledge the limitations of this study. It was conducted retrospectively with manual data extraction and entry, which introduces the possibility of data entry errors and patient selection bias. Although this was a single-center study, all consecutive patients treated with ICIs during the specified period were included, minimizing the potential for selection bias. The relatively small sample size and limited number of events in our cohort prevented comprehensive multivariable analyses and draw definitive conclusions.

In conclusion, the strong correlation between high pre-treatment levels of sATP^CD4^ and nATP^CD4^ with irAEs in advanced NSCLC patients undergoing ICIs suggests their potential as useful indicators. These findings may aid in counseling patients prior to initiating immunotherapy. However, further prospective studies with larger sample sizes and multi-center participation are necessary to validate our results.

## Conclusion

Our study revealed that peripheral blood WBC, nATP^CD4^, and sATP^CD4^ concentrations were associated with an increased risk of irAEs in advanced NSCLC patients treated with ICIs. The optimal cutoff values for WBC, nATP^CD4^ and sATP^CD4^ as 8.165 × 10^9^ cells/L, 484.5 ng/mL, and 156 ng/mL, respectively. These findings provide potential peripheral blood markers for identifying patients at higher risk of irAEs during ICI therapy. Further validation and expansion of these results are warranted.

## Data Availability

The raw data generated and analyzed during the current study are available from the corresponding author on reasonable request.
